# Global supermarkets’ corporate social responsibility commitments to public health: a content analysis

**DOI:** 10.1186/s12992-018-0440-z

**Published:** 2018-11-29

**Authors:** Claire Elizabeth Pulker, Georgina S. A. Trapp, Jane Anne Scott, Christina Mary Pollard

**Affiliations:** 1 School of Public Health, Curtin University, Kent Street, GPO Box U1987, Perth, WA 6845 Australia; 2 Telethon Kids Institute, The University of Western Australia, PO Box 855, West Perth, WA 6872 Australia; 3 School of Population and Global Health, The University of Western Australia, 35 Stirling Highway, Crawley, WA 6009 Australia; 4 East Metropolitan Health Service, Kirkman House, 20 Murray Street, East Perth, WA 6004 Australia

**Keywords:** Supermarket, Corporate social responsibility, CSR, Globalization, Public health, Nutrition, Sustainability

## Abstract

**Background:**

Supermarkets have unprecedented political and economic power in the food system and an inherent responsibility to demonstrate good corporate citizenship via corporate social responsibility (CSR). The aim of this study was to investigate the world’s largest and most powerful supermarkets’ publically available CSR commitments to determine their potential impact on public health.

**Methods:**

The world’s largest 100 retailers were identified using the *Global Powers of Retailing* report. Thirty-one supermarkets that published corporate reports referring to CSR or sustainability, in English, between 2013 and 2018, were included and thematically analysed.

**Results:**

Although a large number of themes were identified (*n* = 79), and there were differences between each business, supermarket CSR commitments focused on five priorities: donating surplus food to charities for redistribution to feed the hungry; reducing and recovering food waste; sustainably sourcing specific ingredients including seafood, palm oil, soy and cocoa; governance of food safety; and growing the number of own brand foods available, that are made by suppliers to meet supermarkets’ requirements.

**Conclusions:**

CSR commitments made by 31 of the world’s largest supermarkets showed they appeared willing to take steps to improve sustainable sourcing of specific ingredients, but there was little action being taken to support health and nutrition. Although some supermarket CSR initiatives showed promise, the world’s largest supermarkets could do more to use their power to support public health. It is recommended they should: (1) transparently report food waste encompassing the whole of the food system in their waste reduction efforts; (2) support healthful and sustainable diets by reducing production and consumption of discretionary foods, meat, and other ingredients with high social and environmental impacts; (3) remove unhealthful confectionery, snacks, and sweetened beverages from prominent in-store locations; (4) ensure a variety of minimally processed nutritious foods are widely available; and (5) introduce initiatives to make healthful foods more affordable, support consumers to select healthful and sustainable foods, and report healthful food sales as a proportion of total food sales, using transparent criteria for key terms.

## Background

Globally, the proportion of foods sourced from supermarkets has increased [1]. A global ‘supermarket revolution’ has been taking place for the last 30 years, with phenomenal growth in supermarket sales in developing countries [2]. In 2017, IPES-Food reported that a third of global food sales were made by the ten largest supermarket chains [3], which highlights the important role of supermarkets in global food provision. The increase in supermarket food sales in developing countries has been at the expense of more traditional outlets, and is associated with dietary changes that may impact public health [1, 4]. For example, supermarkets tend to sell a wider variety of highly processed foods compared to traditional retailers, which can contribute to poor diets and increases in population overweight and obesity [1, 4].

### Supermarket power and influence

Supermarkets have been described as having unprecedented and disproportionate power in the global food system [1]. A review of the sources of supermarket power in Australia identified them as being the primary gatekeepers of the food system [5]. Whilst companies from other sectors of the food industry, including food manufacturers, food service operators, and their industry associations, also wield political power [6], their influence over public policy compared to supermarkets has not been explored [5], and supermarkets are the focus of this study. Some large corporations such as supermarkets have greater economic power than governments [7]. In fact, some of the world’s biggest corporations make more money than many countries [8]. Using financial data from 2015, supermarket chain Walmart ranked as the tenth largest global economy, higher than Australia at twelfth; and the top 250 global economies included nine supermarket chains [8]. With such great political and economic power, the relationship between corporation and society becomes critical.

One of the most important consequences of supermarket domination of the food system is growth in supermarket own brand foods [9]. Supermarket own brand foods (also known as private label, in-house brand, store brand, retailer brand, or home brand) are owned by retailers, wholesalers or distributors and are sold privately in their own stores [10], which means they have a dual role in manufacturing and retailing. There has been rapid development and global expansion of supermarket own brand foods [11, 12]. For example, in the UK, Spain and Switzerland, supermarket own brands account for up to 45% of national grocery sales [11]. The products can be sourced globally, so there is less dependence on local suppliers [13], enabling increased supermarket control over supply chains for greater returns [9].

### Supermarket corporate social responsibility

The neoliberal political context favoured by large multi-national corporations aims to minimise the regulatory role of government in order to promote free trade [14]. This assumes market forces will establish the best outcomes for society. Supporters of the approach say voluntary corporate actions are lower cost, more flexible, and less adversarial than traditional regulatory approaches [15]. In response to concerns for the environment, in 1987 the United Nations (UN) called for a global agenda for change which considered the relationships between people, resources, environment and ongoing development [16]. The UN World Commission on Environment and Development suggested large corporations could do more to address this challenge [16].

Corporations have attempted to manage their impact on the world’s resources and communities by implementing corporate social responsibility (CSR) strategies. These voluntary measures have been framed by food companies as socially responsible initiatives designed to ensure consumer welfare [17], however, CSR has been criticized as a means for food companies to prevent regulation [18], or place responsibility for selecting healthy foods onto consumers [19]. At the same time, CSR has been described as a source of structural power, whereby supermarkets are able to use CSR to set limits on the range of choices available to other food system actors (e.g. growers, manufacturers, consumers) by agenda-setting and rule-setting [20]. For example, Australian supermarkets have used CSR to exert control over farmers and growers by stipulating environmental management practices that must be met to achieve supplier status [5]. It has also been asserted that government regulation is the only effective mechanism to prevent the public harm caused by unhealthy food, because the purpose of corporations is to maximise profit [21].

Whilst there is no agreed definition of CSR, Garriga and Melé (2004) have mapped the theories and approaches in a conceptual framework that includes: (i) instrumental, (ii) ethical, (iii) integrative, and (iv) political theories [7]. Instrumental theories describe CSR as a means to generate profits; ethical theories understand CSR as an ethical obligation of corporations to society; integrative theories argue that CSR is required because corporations rely on society for continued success; and political theories state that the power held by large corporations demands they act responsibly via CSR [7]. The main difference between the CSR theories which have been mapped in the conceptual framework is the apparent corporate motivation.

For the purpose of this study the political CSR lens is applied, whereby powerful supermarkets have an inherent responsibility to society, particularly when neo-liberal governments fail to protect their citizens [7]. Political CSR theories include ‘corporate constitutionalism’, which states that corporate power is limited by constituency groups within society, who demand corporations act responsibly; and if their power isn’t used to benefit society it will be lost [7]. ‘Corporate citizenship’ is another political CSR theory which describes corporations as belonging to a community, which they need to take account of by acting responsibly, and addressing global challenges [22]. The political CSR lens does not include analysis of ‘corporate political activity’, which investigates the ways corporations attempt to influence political outcomes that can influence public health, for example by lobbying or using legal action [23].

### Evaluation of CSR efforts

Assessment of CSR using a political lens is important to hold large companies, including food retailers, to account and a number of initiatives currently undertake this task. The political CSR approach is evident in the Access to Nutrition Index (ATNI) assessment of global food manufacturers’ CSR impact on public health [24]. The ATNI aims to encourage food companies to make healthy products more accessible, and influence consumers’ food choice and behaviour responsibly [24]. The ATNI has also garnered support from global investors, who have committed to factor the nutrition practices of food corporations into their investment decisions [25]. Despite the global proliferation of supermarket own brands [12], they are not currently included within the ATNI’s scope. The International Network for Food and Obesity/Noncommunicable Diseases Research, Monitoring and Action Support (INFORMAS) aims to standardise the monitoring of food environments in diverse countries and settings [26]. Food environments, also referred to as nutrition environments, include the settings (e.g. home, school, workplace, and food retail outlets including supermarkets and restaurants) that provide access to food [27]. INFORMAS have developed a country-level supermarket assessment tool to rate CSR policies and commitments related to obesity prevention and nutrition, based on the ATNI methods [28]. Analysis of Australian supermarkets recommends they take much stronger action [29].

Global reporting initiatives, including the FTSE4Good index [30] and the Dow Jones Sustainability index [31], encourage responsible corporate practices by reporting on performance to global investors. The UN Global Compact, which corporations can sign up to, encourages CSR by setting out ten guiding principles which cover human rights, labour, the environment, and anti-corruption [32]. In France, the *Grenelle Acts* enforced annual CSR reporting by large companies on 40 topics related to managing their social and environmental impact, and commitments to sustainable development [33]. The Global Reporting Initiative (GRI) Sustainability Reporting Guidelines provide a reference for disclosure of the environmental, social and economic impacts of global organisations, to achieve transparency in CSR reporting, and recommend corporate reports should reflect both positive and negative aspects of performance to provide balance [34]. The EAT-Lancet Commission, established to scientifically assess the changes needed to deliver healthy sustainable diets, will report on which companies control the global food system and whether change is considered possible [35].

To date, there have been few investigations of supermarket CSR commitments to public health internationally. Peter et al. (2007) studied the CSR activities of the top ten global food retailers, finding that only five supermarkets produced dedicated CSR reports [36]. Examination of CSR commitments to healthy eating by the largest supermarkets in the UK in 2005 concluded that they could do more to support their customers [37]. Despite being a nutrition initiative, the primary motivation for removing confectionery from prominent in-store locations was to achieve competitive advantage by appealing to customers [38]. Souza-Monteiro et al. (2017) analysis of UK supermarkets’ CSR concluded it still appeared to be used as a tool for competition [39]. A US study of CSR commitments by the country’s top 100 retailers revealed that food retailers, including supermarkets and restaurants, had the highest proportion of CSR content on their websites [40]. Their focus tended to be on social and environmental initiatives, such as sponsorship of local community charities and projects [40]. The examples illustrate the marked differences in the nature and content of supermarket CSR, with CSR activity rarely occurring at the expense of commercial priorities [36].

Supermarket CSR commitments to protect public health should encompass managing a healthy and sustainable food supply, including taking responsibility for food waste. Analysis of publically available CSR commitments to reducing waste by the top ten US supermarket chains has recently been conducted [41]. Comparisons were made with Tesco in the UK, which was used as an exemplar. Tesco were commended for extending their food waste efforts throughout the supply chain, tracking and reporting on progress, and focusing on prevention and partnerships [41]. In comparison, all but one US supermarket, Ahold Delhaize, failed to transparently report food waste and only four had food waste reduction commitments [41].

The significant power of the world’s largest supermarkets is likely to have many implications for public health. For example, Australian supermarkets were found to exert influence in three key domains, namely food governance (i.e. how rules or decisions about food are made), the food system (i.e. livelihoods and communities), and public health nutrition (i.e. determinants of health) [5]. Food environments including supermarkets have been identified as a driver of poor diet [26, 42, 43], which is one of the most important risk factors for early deaths globally [44]. However, public health-led interventions in supermarket settings can lead to increased purchases of healthy foods [45, 46]. They have the power to create food environments supportive of healthy food choices, which UK supermarkets have publically acknowledged [47]. What is missing is an assessment of the CSR activity of the world’s largest and most powerful supermarkets, to understand where progress is being made on protecting public health, and the improvements needed. Critique of supermarkets’ CSR has the potential to stimulate change throughout the food system [48].

To date, there has not been a systematic analysis of global supermarket CSR commitments to protect public health. There is a significant gap in knowledge about how supermarkets address the global challenge of supporting and encouraging healthy and sustainable diets. This study aimed to investigate publically available CSR commitments that impact public health by the world’s largest and most powerful supermarkets.

## Methods

### Study scope

The specific research question was: What public health related CSR commitments have been made by supermarket chains globally? This analysis focused on CSR commitments related to food and non-alcoholic beverages in the three domains of food governance, the food system, and public health nutrition. Food governance CSR commitments describe rules or decisions that impact the food system [49]. Food system CSR commitments impact the people whose livelihoods depend upon making food available, including farmers and food manufacturers, and their communities [50]. CSR commitments to public health nutrition impact the provision of safe, nutritious, affordable, secure, and environmentally sustainable food [51].

Supermarkets’ CSR activity to reduce the environmental impact of buildings and distribution networks, and minimise harm from alcohol, tobacco, gambling, or other business interests were excluded. These initiatives are an important way for supermarkets to reduce their impact on people and the planet, but are beyond the scope of this review due to the focus on how supermarkets can support and encourage healthy and sustainable diets.

### Selection of companies

INFORMAS, which aims to standardise food environments monitoring in diverse countries and settings [26], recommends focusing on predominant food outlet types [52]. Therefore, the focus of this study was commitments made by the largest supermarket chains worldwide to support and encourage healthy and sustainable diets.

The world’s largest one hundred retailers (of all types) were identified using the 2018 *Global Powers of Retailing* report [53]. Compiled annually by auditor Deloitte, this report ranked retailers using publically available information for the financial year ending in June 2017. The largest 100 retailers comprised 44 supermarket chains, hypermarket chains, and discount supermarket chains (referred to simply as supermarkets henceforth), which were selected for this study. The Fortune 500 report was not used as the tool for selecting the world’s largest supermarkets, as it only considers companies that are incorporated and operate in the US [54].

### Data collection

Websites for each of the selected supermarkets were searched for company reports referring to CSR or sustainability. The GRI’s Sustainability Disclosure Database (GRI database) [34] was also searched to identify whether reports had been lodged by the supermarkets, and whether they were in the recommended format (i.e. GRI-G4). Reports in languages other than English were excluded for practical reasons (13 reports). Corporate reports that referred to CSR or sustainability were identified. For each included supermarket, information about the dominant retail format (e.g. discount store, hypermarket), country of origin, annual retail revenue, the number of countries where they operate, and the number of supermarkets were recorded. Participation in the GRI database, and presence on the Fortune 500 list were also recorded. Supermarket reports referring to CSR or sustainability provided the research materials for this study.

Supermarket reports had a number of different names assigned by the corporations, including: global responsibility report, sustainability report, corporate responsibility report, annual activity and responsible commitment report, sustainable retailing performance, green mission report, and corporate citizenship report. In addition, CSR was referred to within some annual reports. Separate CSR commitments or strategies were published by some supermarkets, and these were included as research materials.

### Theoretical framework applied

**Fig. 1:**
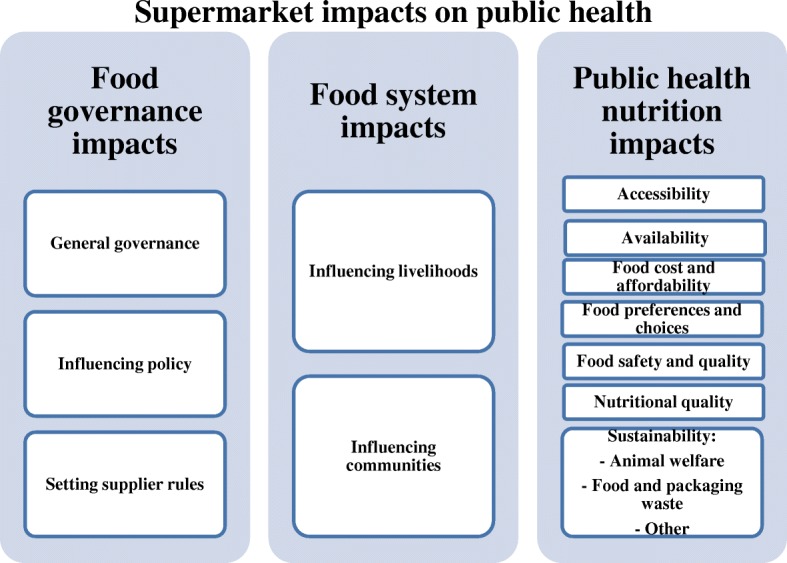
Framework of supermarket impacts on public health, based on evidence of how supermarket power impacts public health [5]; it includes three domains and 14 attributes

A framework was developed to analyse the CSR reports based on evidence of how supermarket power impacts public health [5] (Fig. 1). For this study, content analysis of CSR reports identified themes relating to the following 14 attributes: general governance, influencing policy, setting supplier rules, influencing livelihoods, influencing communities, accessibility, availability, food cost and affordability, food preferences and choices, food safety and quality, nutritional quality, animal welfare, food and packaging waste, and other sustainability issues.

### Data analysis

Supermarket reports were entered into NVivo11 and the first author reviewed them for content relating to the theoretical framework, with each segment of coded text referred to as a ‘CSR statement’. The process included initial familiarisation with the reports, followed by coding selected text to the 14 attributes listed above. Each of the coded text segments was reviewed again for important themes.

## Results

Thirty-one supermarkets met the inclusion criteria for this study, i.e. a supermarket listed in the top 100 retailers (of all types) in the 2018 *Global Powers of Retailing* report [53], with a CSR or sustainability report available in English. The list includes five companies listed on the Fortune 500 list (Table 1). Supermarket countries of origin included Germany, France, the Netherlands, the UK, Switzerland, Spain, Italy, Sweden, Finland, the US, Canada, Australia, South Korea, Chile, South Africa, and Hong Kong. Six of the companies only operated in one country (the US, the UK or Canada) and the rest operated in between two and 50 countries. For example, US-based Walmart operated supermarkets in 27 countries including Argentina, Canada, Ghana, China, India, Japan, and Uganda; Netherlands-based Ahold Delhaize operated supermarkets in 11 countries including the US, Belgium, Greece, and Romania; South Korea-based Lotte Shopping operated supermarkets in six countries including China, Indonesia, and Russia. The number of supermarket outlets ranged from 245 (Hy-Vee Inc) to 6548 (Dairy Farm International Holdings Ltd). Most (24/31) supermarkets participated in the GRI database, however only 12 reports were compliant with the GRI-G4 standard.

**Table 1:** Summary of the world’s largest supermarkets based on data sourced from *Global Powers of Retailing* [53]

FY2016 Retail revenue rank	Name of company	Dominant retail format	Country of origin	FY2016 Retail revenue (US$M)^a^	Number of countries	Number of supermarkets	Main supermarket chain(s)	Participate in the GRI Sustainability Disclosure Database^b^	Type of report (as per GRI database)	Fortune 500 company	Title of reviewed report (year), and website link for non-GRI reports
1	Wal-Mart Stores, Inc.	Hypermarket/ Supercentre/ Superstore	US	485,873	29	11,700	Walmart, Sam’s, Club Massmart, Asda	Yes	GRI-G4	#1	Global responsibility report (2017)
3	The Kroger Co.	Supermarket	US	115,337	1	2796	Kroger, Ralphs, Dillons, Smith’s	Yes	Non-GRI	#18	Sustainability report (2017)
8	Aldi Einkauf GmbH & Co. oHG	Discount Store	Germany	84,923	17	10,132	Aldi	Yes	North: Non-GRI; South: GRI-G4	–	Aldi North Group: Sustainability report (2015), Aldi South Group: International corporate responsibility report (2015)
9	Carrefour S.A.	Hypermarket/ Supercentre/ Superstore	France	84,131	34	11,935	Carrefour, Champion	Yes	GRI-G4	–	Annual activity and responsible commitment report (2016)
11	Tesco PLC	Hypermarket/ Supercentre/ Superstore	UK	72,390	8	6809	Tesco	Yes	Non-GRI	–	Annual report and financial statements (2017), Little Helps Plan (2017)
14	Ahold Delhaize	Supermarket	Netherlands	68,950	11	6556	Delhaize, Albert Heijn Food Lion, Hannaford	Yes	GRI-G4	–	Supplementary report on sustainable retailing performance (2016), Annual report (2016)
17	Albertson’s Companies, Inc.	Supermarket	US	59,678	1	2300	Albertsons, Safeway Tom Thumb	No	–	#49	Sustainability update (2016) https://www.albertsons.com/our-company/social-responsibility/
18	Auchan Holding SA	Hypermarket/ Supercentre/ Superstore	France	57,219	14	3778	Auchan, Jumbo Alcampo, Simply, Market	Yes	Non-GRI	–	CSR section of the 2016 management report (2016)
21	Wesfarmers Limited	Supermarket	Australia	47,690	4	801	Coles	Yes	GRI-G4	–	Sustainability report (2017)
22	Rewe Group	Supermarket	Germany	44,641	11	14,728	Rewe, Penny, Adeg	Yes	GRI-G4	–	Sustainability report (2015/16)
23	Woolworths Limited	Supermarket	Australia	40,773	3	1179	Woolworths, Countdown	Yes	GRI-G4	–	Corporate responsibility strategy 2020 (2017), Annual report (2017), Corporate social responsibility report (2017)
24	Casino Guichard-Perrachon S.A.	Hypermarket/ Supercentre/ Superstore	France	39,856	27	12,969	Casino, Franprix Leader Price, Libertad Pão de Açúcar	No	–	–	Annual and corporate social responsibility performance report (2016) https://www.groupe-casino.fr/en/wp-content/uploads/sites/2/2017/06/RA-2016-GB.pdf
29	Publix Super Markets, Inc.	Supermarket	US	34,274	1	1182	Publix	Yes	Non-GRI	#85	Sustainability report (2017)
30	Loblaw Companies Limited	Hypermarket/ Supercentre/ Superstore	Canada	34,235	6	2300	Loblaws, Zehrs, Provigo	Yes	Non-GRI	–	Corporate social responsibility report (2016)
31	J Sainsbury plc	Supermarket	UK	34,048	2	1200	Sainsbury’s	Yes	Non-GRI	–	20 × 20 sustainability plan (2014), Annual report and financial statements (2017)
39	Migros-Genossenschafts Bund	Hypermarket/ Supercentre/ Superstore	Switzerland	24,152	3	685	Migros	Yes	GRI-G4	–	Annual report (2016)
40	Lotte Shopping Co., Ltd.	Hypermarket/ Supercentre/ Superstore	South Korea	23,991	6	886	Lotte Mart	Yes	GRI-G4	–	Annual report (2016), Sustainability report (2014)
43	Coop Group	Supermarket	Switzerland	28,744	7	2295	Coop, Sapori d’Italia The Karma, shop	Yes	Cites GRI	–	Sustainability progress report (2016)
47	Mercadona, S.A.	Supermarket	Spain	21,905	2	1574	Mercadona	No	–	–	Satisfying “The Boss” Annual report (2015) https://info.mercadona.es/en/who-we-are/press-hall/annual-reports
48	Wm Morrison Supermarkets PLC	Supermarket	UK	21,744	1	491	Morrisons	Yes	Non-GRI	–	Corporate responsibility report (2016/17)
53	Empire Company Limited	Supermarket	Canada	18,065	1	1500	Sobeys	No	–	–	Annual report (2016) https://www.empireco.ca/wp-content/uploads/2017/03/Empire-AR-2016_ENG-FINAL-SEDAR.pdf
59	Whole Foods Market, Inc.	Supermarket	US	15,724	3	481	Whole Foods, Market	Yes	Non-GRI	#176	Green mission report (2012)
64	Cencosud S.A.	Supermarket	Chile	14,525	5	384	Jumbo, Gbarbosa Santa Isabel, Wong, Metro	Yes	Non-GRI	–	Annual memory (2016)
67	Marks and Spencer Group plc	Department store/ Supermarket	UK	13,837	50	1025	Marks and, Spencer	Yes	GRI-G4	–	Plan A report (2017), Plan A 2025 commitments (2017)
70	John Lewis Partnership plc	Department store/ Supermarket	UK	13,361	6	352	Waitrose	Yes	Non-GRI	–	Sustainability report (2016), Annual report and accounts (2017)
78	Conad Consorzio Nazionale, Dettaglianti Soc. Coop. a.r.l.	Supermarket	Italy	12,345	2	2673	Conad, Margherita, Todis, Sapori &, Dintorni	No	–	–	Annual report (2015) https://en.calameo.com/read/001456897077b0490e97a
80	ICA Gruppen AB	Supermarket	Sweden	11,824	5	1300	ICA, Rimi	Yes	GRI-G4	–	Sustainability report: Jan-March, Apr-June, Jul-Sept (2017), Annual report (2017)
85	Dairy Farm International Holdings Limited	Supermarket	Hong Kong	11,201	11	6548	Wellcome, Yonghui, Cold Storage, Jasons, Marketplace Giant	No	–	–	Annual report (2016) https://www.dairyfarmgroup.com/en-US/Investors/Financial-Reports
88	S Group	Supermarket	Finland	10,835	5	1633	S Market, Prisma, Alepa, Sale	Yes	GRI-G4	–	Responsibility report (2016)
94	Shoprite Holdings Ltd.	Supermarket	South Africa	10,340	15	2689	Shoprite, Usave, Checkers	Yes	Non-GRI	–	Integrated report (2017)
99	Hy-Vee, Inc.	Supermarket	US	9800	1	245	Hy-Vee	No	–	–	Corporate citizenship report (2017) https://www.hy-vee.com/corporate/our-company/corporate-citizenship-report

Supermarket CSR reports addressed 79 themes (listed 1–79 in Table 2) across the 14 attributes included in the theoretical framework (Fig. 1). Most (57/79) themes related to public health nutrition, followed by food governance (10/79), and then food system (12/79) themes. Table 2 provides details of the CSR themes reported across all supermarkets. Table 3 summarises the CSR commitments made by each supermarket, cross-referenced with the themes reported in Table 2 that were included in the publically available reports.

**Table 2:** Thematic analysis of supermarket corporate social responsibility commitments that impact public health

*Food governance*							
*General governance*	(1) Participates in global governance initiatives, e.g. GRI database, Dow Jones Sustainability Index, Global Compact	(2) Aims to improve population nutrition and health	(3) Upholds ethical practice by a code of conduct or similar				
*Influencing policy*	(4) Participates in government-led public health nutrition initiatives	(5) Works with key influencers on setting food, nutrition, or sustainability standards and policies	(6) Is transparent about relationships including with external groups, and own brand suppliers				
*Setting rules for suppliers*	(7) Requires third party quality accreditation, e.g. Global Gap	(8) Sets standards for producers of supermarket own brand products	(9) Sets other private standards for suppliers	(10) Sets rules for social and environmental issues			
*Food system*							
*Influencing livelihoods*	(11) Sources local food products	(12) Pays food producers a fair price and/or has fair payment terms	(13) Pays staff a fair wage, and/or provides healthy working conditions	(14) Deals with suppliers in an ethical way	(15) Provides financial assistance e.g. loans, or training to small/ local businesses	(16) Promotes local or regional foods in other countries	
*Influencing communities*	(17) Highlights charitable food donations made	(18) Makes food donations for animals	(19) Provides other support to food charities e.g. infrastructure, training	(20) Supports community organisations via provision of space and other resources	(21) Provides community support via funding specific food and nutrition projects	(22) Provides emergency aid to communities or staff affected by natural disasters	
*Public health nutrition*							
*Accessibility*	(23) Considers the location of stores in communities	(24) Considers the location of foods in stores e.g. removes unhealthy foods from prominent locations	(25) Provides consumer education initiatives to support healthy eating, e.g. store tours, menu planning, cooking skills	(26) Provides consumer education initiatives related to sustainability, e.g. ways to reduce food waste, animal welfare information	(27) Has promotions to encourage sales of healthy foods	(28) Increases accessibility of supermarket own brands by making them available to other retailers or other countries	
*Availability*	(29) Sells healthy foods	(30) Sells sustainable foods	(31) Sells locally sourced or regional foods	(32) Sells fresh food	(33) Sells products to meet specific needs	(34) Sells supermarket own brand products	(35) Sells convenient products
*Food cost and affordability*	(36) Offers foods that are affordable	(37) Ensures healthy foods are no more expensive than unhealthy foods	(38) Tracks shopping basket affordability via ongoing monitoring	(39) Offers foods that meet specific needs at a competitive price	(40) Keeps the cost of supermarket own brand products down	(41) Offers discounts or subsidies on healthy foods, or other foods that meet specific needs	
*Food preferences and choices*	(42) Has food labelling initiatives to enable consumers to identify healthy and/or sustainable foods	(43) Has food labelling initiatives to enable consumers to identify foods that meet specific needs, e.g. free from, vegetarian	(44) Has food labelling/ marketing initiatives to identify locally sourced or regional products, e.g. logos, catalogues	(45) Has food labelling/ marketing initiatives related to animal welfare	(46) Highlights healthier food choices using in-store signage e.g. shelf edge labels	(47) Highlights healthier food choices on shopping websites	(48) Highlights sustainability messages e.g. minimise food waste, recycle food packaging
*Food safety and quality*	(49) Makes food safety statements	(50) Makes statements about food quality	(51) Emphasises traceability	(52) Ensures hygienic stores	(53) Avoids use of artificial ingredients, e.g. colours, flavours, preservatives, BPA-free packaging	(54) Avoids use of genetically modified ingredients	
*Nutritional quality*	(55) Has a nutrient reduction programme for supermarket own brand foods	(56) Has specific healthy food ranges	(57) Has established targets for healthy foods to contribute a significant proportion of total food sales	(58) Has established targets to improve the overall nutritional profile of foods sold	(59) Has established targets to reduce portion sizes of single serve snacks		
*Sustainability - animal welfare*	(60) Encourages sustainable fishing practices	(61) Minimises use of hormones or antibiotics	(62) Upholds the five freedoms of animals to ensure their welfare	(63) Sells cage-free eggs	(64) Sets standards for dairy cow welfare	(65) Has other initiatives to improve animal welfare	(66) Bans products from sale due to animal welfare concerns
*Sustainability - food and packaging waste*	(67) Has established targets to reduce food waste	(68) Sells imperfect fresh produce, or uses it to make meals or products	(69) Has established targets to reduce waste in the whole of the food system	(70) Has established targets to reduce and recycle packaging waste	(71) Sources packaging materials from sustainably managed forests	(72) Has established targets to reduce waste by moving paper-based marketing materials e.g. coupons, to digital formats	
*Sustainability - other*	(73) Sustainably sources coffee	(74) Sustainably sources cocoa	(75) Sustainably sources palm oil	(76) Sustainably sources soy	(77) Sustainably sources other ingredients	(78) Sources organic products	(79) Has other product related sustainability commitments

**Table 3:** Summary of the world’s largest supermarkets’ corporate social responsibility commitments that impact public health

Name of company	Food governance	Food system	Public health nutrition						
			Accessibility	Availability	Food cost and affordability	Food preferences	Food safety and quality	Nutritional quality	Sustainability
Wal-Mart Stores, Inc. (*n* = 37)	2, 4, 5, 9, 10	11, 13, 15, 17, 19, 22	23, 25, 26	–	36, 41	42, 47	49, 51, 53	55, 56	60, 61, 62, 63, 64, 65, 67, 68, 69, 70, 75, 76, 77, 79
The Kroger Co. (*n* = 35)	1, 2, 3, 4, 5, 6, 8, 9, 10	11, 13, 14, 17, 22	–	33, 34	–	43, 44, 45	49, 50, 52, 53	–	60, 61, 63, 64, 65, 66, 67, 70, 72, 73, 75, 79
Aldi Einkauf GmbH & Co. oHG (*n* = 29)	7, 9	11, 13, 17, 21	24, 25, 26	32, 33, 34	–	42, 43, 44, 45	50, 53, 54	55	60, 62, 65, 66, 70, 73, 74, 75, 79
Carrefour S.A. (*n* = 30)	1, 2, 8	11, 12, 17, 19, 21, 22	23, 27, 28	31, 33, 34, 35	36, 39, 40	43	49, 50, 51	–	60, 61, 67, 69, 75, 77, 79
Tesco PLC (n = 29)	1, 2	12, 13, 17, 20, 21	24	34	36, 37, 40, 41	46, 47	49	55, 58	60, 64, 65, 67, 68, 69, 70, 74, 75, 77, 79
Ahold Delhaize (*n* = 31)	1, 2, 3, 7, 8	13, 17, 18	25	33, 34	36	42, 43, 45, 46	49, 50	55, 57	60, 64, 66, 67, 68, 69, 73,74, 75, 76, 77
Albertson’s Companies, Inc. (*n* = 6)	–	17, 22	25	34	–	–	–	–	70, 75
Auchan Holding SA (n = 29)	1	11, 13, 15, 16, 17, 18, 19, 20, 21	24, 25, 27	29, 33, 34	36	42, 43, 45	49, 51, 52, 54	–	60, 67, 70, 75, 79
Wesfarmers Limited (*n* = 28)	1, 4, 8, 9	11, 12, 13, 15, 17, 22	–	32	36	42	49, 51, 53	55	60, 62, 63, 65, 67, 68, 70, 73, 74, 75, 77
Rewe Group (*n* = 34)	1, 3, 5, 8, 9	13, 17	25	30, 21, 33, 34	–	42, 43, 44, 45, 47	49, 50	–	60, 62, 63, 64, 65, 66, 67, 68, 70, 74, 75, 76, 77, 78, 79
Woolworths Limited (*n* = 32)	1, 4, 7, 8, 9, 10	11, 14, 17, 21, 22	24	–	36, 38	42, 45, 47, 48	53	55	60, 63, 64, 65, 67, 68, 69, 70, 73, 74, 75, 77
Casino Guichard-Perrachon S.A. (n = 30)	2, 3, 7, 8	11, 12, 17	23, 25, 28	33, 34	36, 39, 40, 41	44	49	55, 57	60, 61, 63, 64, 65, 68, 70, 75, 77, 79
Publix Super Markets, Inc. (*n* = 12)	3	13, 17, 18	–	34	–	46	–	–	60, 61, 62, 65, 67, 70
Loblaw Companies Limited (*n* = 22)	2, 5	17, 19, 22	25	33, 34	–	46, 47	49, 51	–	60, 61, 63, 64, 65, 68, 70, 74, 75, 77
J Sainsbury plc (*n* = 25)	2, 3, 10	11, 13, 17, 18	25	33, 34	36	42, 43, 47	–	55, 56, 57	61, 63, 65, 67, 69, 70, 75, 77
Migros-Genossenschafts Bund (*n* = 24)	2, 7, 8	13, 17, 23	25	33	–	42, 43, 44	49	55	60, 65, 66, 67, 70, 73, 74, 75, 76, 77, 79
Lotte Shopping Co., Ltd. (*n* = 16)	1, 3, 9	11, 12, 13, 15, 21	–	–	41	44	49, 51, 52	–	67, 70, 72
Coop Group (*n* = 26)	1, 7, 8, 9	17, 22	23, 26	30, 31, 33, 34	–	44, 45	51	–	60, 63, 64, 65, 70, 73, 74, 75, 76, 77, 79
Mercadona, S.A. (*n* = 13)	1, 4, 5	11, 12, 13, 17	–	33, 34	36	–	49	–	68, 79
Wm Morrison Supermarkets PLC (n = 28)	2, 3, 4, 5, 8, 9	12, 14, 17, 20	–	–	41	42, 43, 46, 47	49	55, 56, 57	60, 61, 63, 67, 68, 69, 70, 75, 76
Empire Company Limited (*n* = 19)	–	11, 17, 19, 21	24, 25	33, 34	36	45	49	–	63, 65, 67, 68, 69, 70, 77, 79
Whole Foods Market, Inc. (n = 22)	8, 10	15, 17, 20, 21	25	29, 33	–	43, 45	50, 54	–	60, 61, 62, 65, 67, 70, 73, 78
Cencosud S.A. (*n* = 10)	1, 3, 6, 9, 10	11, 13, 14, 17	–	–	–	44	–	–	–
Marks and Spencer Group plc (n = 26)	1, 5, 8, 9, 10	13, 17, 20, 21	24	33	41	–	53	56, 57, 58, 59	60, 67, 70, 73, 74, 75, 76, 77, 79
John Lewis Partnership plc (*n* = 17)	3, 7, 8	11, 12, 13, 17, 18	25, 28	34	–	–	49	55	60, 64, 75, 76
Conad Consorzio Nazionale, Dettaglianti Soc. Coop. a.r.l. (*n* = 18)	8	11, 12, 16, 17	25	31, 33, 34	36, 39	43, 44, 46	49, 54	–	71, 79
ICA Gruppen AB (n = 31)	1, 2, 3, 4, 5, 8, 9	11, 13, 17, 21	25, 26	29, 30, 31, 34, 35	36	42	49, 50, 52	–	62, 65, 67, 68, 69, 70, 75, 79
Dairy Farm International Holdings Limited (*n* = 4)	–	17	–	34, 35	–	–	49	–	–
S Group (*n* = 14)	2, 3, 5	13, 17	27	30, 32, 33	36	–	–	–	60, 67, 73, 75
Shoprite Holdings Ltd. (n = 17)	8, 9	11, 13, 15, 17, 20, 21, 22	–	29, 34	36, 41	–	49, 52	–	67, 70
Hy-Vee, Inc.(*n* = 5)	–	13	–	–	–	42	–	–	60, 67, 68

Footnote: (1) Participate in global governance initiatives; (2) Aim to improve population nutrition and health; (3) Uphold ethical practice by a code of conduct or similar; (4) Participate in government-led public health nutrition initiatives; (5) Work with key influencers on setting food, nutrition, or sustainability standards and policies; (6) Be transparent about relationships including with external groups, and own brand suppliers; (7) Requires third party quality accreditation; (8) Sets standards for producers of supermarket own brand products; (9) Sets other private standards for suppliers; (10) Set rules for social and environmental issues; (11) Sources local food products; (12) Pays food producers a fair price and/or has fair payment terms; (13) Pays staff a fair wage, and/or provides healthy working conditions; (14) Deals with suppliers in an ethical way; (15) Provides financial assistance or training to small/ local businesses; (16) Promotes local or regional foods in other countries; (17) Highlights charitable food donations made; (18) Makes food donations for animals; (19) Provides other support to food charities; (20) Supports community organisations via provision of space and other resources; (21) Provides community support via funding specific food and nutrition projects; (22) Provides emergency aid to communities or staff affected by natural disasters; (23) Location of stores in communities; (24) Location of foods in stores; (25) Consumer education initiatives on healthy eating; (26) Consumer education initiatives related to sustainability; (27) Promotions to encourage sales of healthy foods; (28) Increases accessibility of supermarket own brands by making them available to other retailers or other countries; (29) Availability of healthy foods; (30) Availability of sustainable foods; (31) Availability of locally sourced or regional foods; (32) Availability of fresh food; (33) Availability of products to meet specific needs; (34) Availability of supermarket own brand products; (35) Availability of convenient products; (36) Offers foods that are affordable; (37) Ensures healthy foods are no more expensive than unhealthy foods; (38) Tracks shopping basket affordability via ongoing monitoring; (39) Offers foods that meet specific needs at a competitive price; (40) Keeps the cost of supermarket own brand products down; (41) Offers discounts or subsidies on healthy foods, or other foods that meet specific needs; (42) Food labelling initiatives to enable consumers to identify healthy and/or sustainable foods; (43) Food labelling initiatives to enable consumers to identify foods that meet specific needs; (44) Food labelling/ marketing initiatives to identify locally sourced or regional products; (45) Food labelling/ marketing initiatives related to animal welfare; (46) Highlights healthier food choices using in-store signage; (47) Highlights healthier food choices on shopping websites; (48) Highlights sustainability messages; (49) Makes food product safety statements; (50) Makes statements about food quality; (51) Emphasises traceability; (52) Ensures hygienic stores; (53) Avoids use of artificial ingredients; (54) Avoids use of genetically modified ingredients; (55) Has a nutrient reduction programme for supermarket own brand foods; (56) Sells healthy food ranges; (57) Established targets for healthy foods to contribute a significant proportion of total food sales; (58) Established targets to improve the overall nutritional profile of foods sold; (59) Established targets to reduce portion size of single serve snacks; (60) Encourages sustainable fishing practices; (61) Minimises use of hormones or antibiotics; (62) Upholds the five freedoms of animals to ensure their welfare; (63) Sells cage-free eggs; (64) Sets standards for dairy cow welfare; (65) Other initiatives to improve animal welfare; (66) Bans products from sale due to animal welfare concerns; (67) Established targets to reduce food waste; (68) Sells imperfect fresh produce, or uses it to make meals or products; (69) Established targets to reduce waste in the whole of the food system; (70) Established targets to reduce and recycle packaging waste; (71) Sources packaging materials from sustainably managed forests; (72) Established targets to reduce waste by moving paper-based marketing materials; (73) Sustainably sources coffee; (74) Sustainably sources cocoa; (75) Sustainably sources palm oil; (76) Sustainably sources soy; (77) Sustainably sources other ingredients; (78) Sources organics; (79) Other product related sustainability commitments

The following results highlight common and less common CSR themes identified. For each key domain, an example of a supermarket CSR commitment is given.

### Food governance

The food governance related theme most commonly reported by the supermarkets referred to setting standards for manufacturers of supermarket own brand products (15/99 food governance CSR statements). For example, Wm Morrison Supermarkets Plc required all own brand suppliers to adhere to their policy of meeting salt targets. Eight supermarkets also set standards for suppliers’ social and environmental performance, including The Kroger Co. which required all suppliers to agree to the vendor code of conduct. The Kroger Co. assessed the risk of human rights violations in the supply chain, and conducted audits for compliance with the code requirements that included child and forced labour, discrimination, environment, ethics, freedom of association, health and safety, subcontracting, working hours and compensation.

Commitments to improving nutrition and health were only stated in reports from 12 supermarkets. Seven supermarkets made statements about working with government to develop and implement public health initiatives, including Australian companies Wesfarmers Ltd. and Woolworths Ltd. who referred to membership of the Healthy Food Partnership, a public-private-partnership initiative led by the Australian government [55].

### Food system

Highlighting charitable food donations was the most commonly reported commitment that impacts the food system, made by all supermarkets apart from Hy-Vee Inc. and Lotte Shopping Co. Ltd. (29/124 food system CSR statements). Supermarkets positioned donation of food not suitable for sale (but safe for consumption) as responsible management of food waste. French supermarkets referred to the country’s legal requirement to donate surplus food (see [56]). American supermarkets referred to the Environmental Protection Agency’s food recovery hierarchy that prioritises feeding hungry people (see [57]). Supermarkets aimed to assist in reducing hunger, and ‘success’ was often measured by the number of meals provided through a supermarket’s contributions. Shoprite Holdings Ltd. operated mobile soup kitchens in addition to making charitable food donations. However, Conad Consorzio Nazionale point out *“Large retail welfare must not and cannot replace the role of institutions, which are in charge of putting solid measures in place to ensure those on low incomes have sufficient food.”*

Six supermarkets supported local charities by providing space and other resources. Tesco Plc made 56 community rooms available for classes and meetings across their UK network of stores. Many Whole Foods Market Inc. stores provided space for farmers markets or served as pick-up locations for community supported agriculture schemes.

Seventeen supermarkets mentioned fair payment for employees. Some referred to exceeding national minimum wages (e.g. J Sainsbury, John Lewis Partnership Plc), whilst others referred to allowing labour representation and collective bargaining (e.g. Shoprite Holdings Ltd., Mercadona SA). Some supermarkets described the efforts they made to support the health and wellbeing of employees.

Although nine supermarkets committed to paying food producers a fair price or fair payment terms, only four supermarkets referred to dealing with suppliers in an ethical way. For example, Wm Morrison Supermarkets Plc and Woolworths Ltd. both referred to membership of the Supplier Ethical Data Exchange which is a web-based system used to share ethical information and reduce auditing requirements for suppliers.

### Public health nutrition

Public health nutrition commitments varied considerably across the supermarkets. Sustainable sourcing initiatives relating to ingredient sourcing (80 CSR statements), animal welfare (79 CSR statements), and reduction of food and packaging waste (69 CSR statements) were most commonly referred to. Nutritional quality (23 CSR statements), food cost and affordability (30 CSR statements), accessibility (35 CSR statements), and food preferences (55 CSR statements) were referred to the least.

#### Accessibility

Consumer education initiatives on healthy eating was most popular theme within accessibility, with 15 supermarkets making commitments (15/35 accessibility CSR statements). For example, Casino Guichard-Perrachon SA had a Responsible Food truck which provided free cooking workshops using recipes to promote a healthy and sustainable diet; and Loblaw Companies Ltd. focused on educating children on how to read food labels and use the Guiding Stars nutrition rating system.

Four supermarkets described consumer education initiatives related to sustainability. For example, Wal-Mart Stores Inc.’s Asda supermarkets in the UK gave consumers advice on food storage and recipes ideas for leftovers, in an effort to reduce food waste.

#### Availability

Twenty supermarkets referred to own brand product availability (20/56 availability CSR statements). The magnitude of some own brand ranges was described, including the organic own brand range from Alberton’s Companies Inc. which was the largest available in the USA. Aldi, which is well known for its focus on own brand products, stated the highest proportion was found in the Belgian and Luxemburg stores at 99.7%. Tesco Plc had developed 2422 supermarket own brand products over the year.

In contrast, only four supermarkets made statements about healthy foods available in their stores, and four supermarkets made statements about sustainable foods. Three supermarkets made statements about available fresh foods.

#### Food cost and affordability

Fifteen supermarkets committed to offering foods that were affordable, the most common commitment within food cost and affordability (15/30 food cost CSR statements). For example, Ahold Delhaize stated *“We want every family in our trading areas to be able to do their weekly shopping with one of our [stores], regardless of their budget, so every supermarket continues to make pricing more competitive.”* Other efforts included Auchan Holding SA’s Russian stores’ commitment to sell some fruits and vegetables below market price so they were affordable to all shoppers. S Group described their commitment to lowering prices as a long-term strategic decision to make shopping affordable. Shoprite Holdings Ltd. described the importance of helping to put food on the table, and said affordability was a key measure of their success.

Three supermarkets committed to offering specific foods at competitive prices. For example, Carrefour SA in Argentina guaranteed the lowest prices for 800 products every day. In addition, three supermarkets made statements about keeping the cost of supermarket own brand products down. Tesco Plc was the only supermarket chain to make a commitment to ensure shoppers always paid the same price or less for healthier options. Woolworths Ltd. was the only supermarket chain to commit to introducing an affordable healthy eating index based on shopper preferences.

#### Food preferences and choices

Statements about food labelling initiatives to enable consumers to identify healthy or sustainable foods were made by 12 supermarkets (12/55 food preferences CSR statements). Seven made statements about assisting consumers to select healthy foods, and five referred to an aspect of sustainability. For example, Australian companies Wesfarmers Ltd. and Woolworths Ltd. had introduced the voluntary Health Star Rating front-of-pack nutrition labelling device on own brand products.

Six supermarkets highlighted healthier food choices in stores using signage: Ahold Delhaize and Loblaw Companies Ltd. used the Guiding Stars system of rating all products available within a store and applied labels on grocery shelves to indicate the healthier choices; and Tesco Plc held a ‘Little Helps to Healthier Living’ event which included ‘Helpful Little Swaps’ signs to highlight products lower in sugar, fat or salt compared to regular alternatives. Products with the ‘Helpful Little Swap’ signs saw a 30% increase in sales during the event.

Seven supermarkets stated they highlighted healthier choices on their shopping websites: Tesco Plc used the ‘Helpful Little Swaps’ campaign; J Sainsbury’s swapping campaign identified lower calorie options; Loblaw Companies Ltd. applied the Guiding Stars system; and Wm Morrison Supermarkets Plc had a dedicated healthier living section which included healthier products.

With the exception of the Australian Health Star Rating algorithm which is publically available, none of the supermarkets provided the criteria used to determine healthy and sustainable foods identified via product labelling, in-store signs, or websites.

#### Food safety and quality

Statements about the importance of food safety were made by 20 supermarkets, with seven making specific traceability commitments (20/49 food safety CSR statements). Most statements referred to the rigorous processes in place to ensure suppliers of supermarket own brand products adhered to the supermarket’s requirements for quality control. Some committed to ensuring all suppliers were compliant with requirements for food safety and correctly labelled products. Third-party assurances were often required from suppliers to demonstrate suitable standards were in place.

#### Nutritional quality

Few supermarkets made commitments to nutritional quality (12/31). Eleven supermarkets committed to nutrient reduction programmes for own brand products (11/23 nutritional quality CSR statements). Targeted nutrients included fat, saturated fat, salt or sodium, sugar, and added sugar, with sugar and sodium receiving the most attention. In addition, Migros-Genossenschafts Bund aimed to increase the fibre content of own brand products. Specific nutrient targets were not provided, with percent reduction, or total amount removed provided by some supermarkets.

Four supermarkets referred to healthy own brand ranges: J Sainsbury’s ‘My Goodness!’ range; Marks and Spencer Group Plc’s ‘Count on Us’ and ‘Balanced for You’ ranges; Wal-Mart Stores Inc’s ‘Great for You’ range; and Wm Morrison Supermarkets Plc’s ‘Eat Smart’ range. Criteria used to determine product healthiness were not disclosed.

Four supermarkets committed to healthy supermarket own brand foods contributing a significant proportion of total food sales. Marks and Spencer Group Plc and J Sainsbury Plc set targets for the contribution of all healthy foods (not just own brand) to total food sales. Criteria used to define healthy foods were not provided.

#### Sustainable sourcing

Commitments to sustainable fishing were made by 22 supermarkets (22/79 animal welfare CSR statements). For example, the sustainable fishing policies of Auchan Holding SA and Aldi Einkauf GmbH & Co. oHG referred to not stocking species that were categorised as endangered or protected. Some supermarkets referred to third party schemes for ensuring the sustainability of the own brand fish sold in their stores, including the Sustainable Fisheries Partnership, Marine Stewardship Council, Aquaculture Stewardship Council, RSPCA Freedom Food, Seafish Responsible Fishing Scheme, WWF Seafood Group, International Seafood Sustainability Foundation, and Sustainable Seafood Coalition.

Commitments to reduce food waste were made by 22 supermarkets (22/69 food and packaging waste CSR statements). Three supermarkets, Ahold Delhaize, J Sainsbury Plc and Tesco Plc, committed to transparently reporting food waste. Tesco Plc had taken this a step further by making a joint commitment with 24 of their largest suppliers to reduce overall food waste across the supply chain. Other food waste reduction initiatives included a partnership between ICA Gruppen AB in Sweden and Karma, a food application, to trial selling food products near their best before date at reduced prices. J Sainsbury replaced multi-buy promotions with lower regular prices to reduce bulk purchasing, which often resulted in wasted food at home. US supermarkets referred to the Environmental Protection Agency’s food recovery hierarchy which prioritises source reduction, followed by feed hungry people, feed animals, industrial uses, composting, with landfill or incineration at the bottom (see [57]).

Supermarket commitments to sustainably sourcing products related to own brand products. Standards referred to include the Roundtable on Sustainable Palm Oil, UTZ Certified, Rainforest Alliance, Fairtrade USA, Fairtrade International, and Bio Suisse. Sustainable sourcing of palm oil was referred to the most, by 21 supermarkets (21/80 sustainable sourcing CSR statements). Ten supermarkets committed to sourcing coffee sustainably. Ten supermarkets referred to sustainably sourcing cocoa, although often this was for specific own brand ranges and did not apply to all products. Eight supermarkets referred to sustainably sourcing soy, which was widely used for animal feed. Fourteen supermarkets referred to sustainably sourcing other ingredients including tea, beef, rice, bananas, fruit juice, hazelnuts, and sugar. Three supermarkets made commitments to sourcing organic products.

## Discussion

Publically available CSR commitments made by 31 of the world’s largest and most powerful supermarkets included 79 themes, identified using a theoretical framework developed by Pulker et al. (2018) to demonstrate how supermarket power impacts public health [5]. Some CSR commitments from some supermarkets indicate they have potential to positively impact public health, but supermarket CSR efforts were generally disappointing.

Although a large number of themes were identified, and there were differences between each business, supermarket CSR commitments consistently focused on the same five priorities. Supermarkets’ efforts to demonstrate good corporate citizenship focused on: (1) donating surplus food to charities for redistribution to feed the hungry; (2) reducing and recovering food waste; (3) sustainably sourcing ingredients including seafood, palm oil, soy and cocoa including via third-party accreditation; (4) governance of food safety including via third-party accreditation; and (5) growing the number of own brand foods available, that are made by suppliers to meet supermarkets’ requirements. These priority themes are described below with real world examples from global supermarkets.

### Donating surplus food to charities for redistribution to feed the hungry

Food charities, such as food banks, provide emergency food relief to people who would otherwise go hungry, and have proliferated in many high-income countries in response to increased food insecurity [58]. To date there is little evidence that charitable food redistribution of unsalable food is an appropriate response for recipients, and researchers challenge the food bank model as a long-term strategy [59]. Concerns have been raised about the ‘industry’ of food banking, described as a business solution that delivers food system efficiency by removing the need for costly landfill [60].

Food donations are essential to food banks, but due to the variability of donated foods nutritional quality cannot be guaranteed [58]. Countries relying on food donations to charities for redistribution to address hunger do not meet human rights obligations, specifically that everyone, regardless of income, has the right to select nutritious and appropriate food in socially acceptable ways [61]. Ironically, many supermarket employees in the US have been found to rely on food assistance such as the Supplemental Nutrition Assistance Program due to low wages, and lack of health care and child care cover [48]. This clearly raises a challenge for supermarkets to provide fair and liveable wages [48].

The powerful supermarkets in this study have reinforced discourse that entwines responsible management of food waste with feeding the hungry. However, charitable food redistribution does not address the underlying structural causes of food insecurity which include poverty, and may even increase inequality [59, 62]. It has been argued that whilst supermarkets continue to support food charities to feed the hungry, governments will not make the social policy reforms needed to ensure citizens’ rights to food are protected [63]. Italy based Conad Consorzio Nazionale were the only supermarket to state that it was the responsibility of the state to support those on low incomes to have sufficient food. Supermarket CSR efforts to feed the hungry should not replace the need for governments to protect the human right to food.

### Reducing and recovering food waste

Food waste is a significant global problem, described as a structural symptom of the ‘broken globalised food system’ [63] (p83). Globally, a third of the food produced is never eaten [64]. Food is wasted throughout the global food system, including from growers, processors, manufacturers, distributors, retailers, food service operators, and end consumers [65]. For example, a UK study showed that most (70%) losses occurred in the home [61].

The World Resources Institute provides companies with guidance on food loss and waste reporting [66]. Committing to reduce food waste throughout the whole of the food system forces supermarkets to address their own practices which contribute to generating waste. These practices include setting cosmetic standards for fresh produce that mean imperfect looking produce is discarded [56]; providing inappropriate packaging formats (e.g. oversized) [67]; encouraging increased food purchases with offers such as ‘buy one get one free’ [68]; or labelling foods with ‘best before’ dates to indicate optimal product quality not required by food regulations [61].

Tesco Plc have been commended for their actions on transparently reporting food waste [41]. They have reported waste profiles for the most commonly purchased foods, including levels and causes, to create tailored waste reduction plans [69]. Recently, they announced removing best before dates from packaging [70]. Only two other supermarkets have committed to transparently reporting food waste, so there is much room for improvement in the scale and impact of global supermarket food waste reduction efforts. Working on solutions that encompass the whole of the food system rather than passing the problem onto other actors is essential [56].

### Sustainable sourcing

Supermarkets in this study consistently framed sustainably sourcing ingredients as the primary method to address sustainable food systems. This included consideration of animal welfare, social, and environmental impacts. Analysis of global food manufacturers found that such sustainable sourcing initiatives overlooked the most important factor, that is how to achieve healthy and sustainable diets [71].

Australian research has evaluated the environmental impact of ‘discretionary’ foods, which are not essential for a healthy diet [72, 73], recommending a reduction in production and consumption as a priority, along with meat reduction, to improve the sustainability of the food system [73]. Discretionary foods are more likely to be ‘ultra-processed’ [74] nutrient-poor industrially processed foods [75]. Dietary guidelines incorporating principles of sustainability recommend avoiding these ultra-processed foods [76].

Although not included in the CSR report, ICA Gruppen in Sweden has taken action to encourage consumers to reduce meat consumption and eat more vegetarian food instead [77]. Supermarkets wishing to make meaningful CSR commitments to support sustainable diets could start by recognising the importance of reducing production and consumption of discretionary foods, meat, and other ingredients with high social and environmental impacts, rather than encouraging ongoing growth from third-party accredited ‘sustainable’ sources.

### Private governance of food safety

The neoliberal political context that minimises regulations in order to promote free trade allows supermarkets to privately govern the food system [5]. The ability to set so called ‘voluntary’ standards for suppliers that must be met is a source of supermarket power that enables control of the supply base [78]. On the other hand however, a major benefit of supermarket private food safety standards is an increasingly safe food supply [5]. Most of the supermarkets in this study focused on assuring safe, correctly labelled foods from all suppliers.

### Growth of supermarket own brand foods

Supermarkets have extended their control over the food system by introducing supermarket own brands. Own brand products offer supermarkets practical benefits, such as flexible global sourcing [79], particularly for shelf-stable processed foods. They can enforce private standards for own brands to manage risk by controlling products, processes, and movement through the supply chain [80]. Globally, market share of supermarket own brands is predicted to grow until they dominate the food supply, led by the largest supermarket chains [81]. Consistent with the literature, supermarkets in this study highlighted their strategies to grow own brand ranges, describing the scale of new product development, strict standards which were often assured by third parties, and the ability to innovate with healthy and sustainable products. Own brand foods offer large global supermarkets the opportunity to positively impact the availability, accessibility, affordability, nutritional quality, product quality, and sustainability of the food supply.

### Gaps in supermarket CSR actions to support public health

Findings show that supermarkets made few CSR commitments to the public health nutrition attributes of accessibility, availability (other than supermarket own brand food development), food cost and affordability, food preferences, and nutritional quality. Whilst supermarkets appeared willing to take steps to improve sustainable sourcing of specific ingredients, there was little action being taken to support health and nutrition. The following section identifies gaps and opportunities.

#### Accessibility

Supermarket CSR initiatives to address accessibility of healthy and sustainable food mainly focused on education. Other CSR initiatives such as ensuring underserved communities had access to supermarkets, and committing to locate nutritious foods in more prominent in-store locations than nutrient-poor foods were less common. The amount of shelf space and the location of foods in stores influence food choice [52]. CSR commitments to remove nutrient-poor confectionery, snacks, and sweetened beverages from checkouts and other prominent areas would assist in protecting public health.

#### Availability

Few CSR commitments were made regarding the public health priority of increasing availability of heathy, sustainably sourced, local, or fresh foods. Instead, supermarket own brand product ranges that meet specific needs such as additive free, vegetarian, organic, and free from common allergens were highlighted. Supermarkets are an important source of healthy foods, however availability is less than ideal: less than half of packaged foods available in Australia and New Zealand could be classified as healthy [82]; household availability of nutrient-poor ultra-processed foods in European countries ranged from 10% in Portugal to 50% in the UK [83]. Ultra-processed foods are increasingly sold in supermarkets around the world [84]. Therefore, ensuring a variety of nutritious fresh or minimally processed foods are widely available in the world’s largest supermarkets is essential for public health.

#### Food cost and affordability

Commitments to ensuring food is affordable were made by a number of supermarkets, however, only two referred to measures that combined cost with health. UK based Tesco Plc stated they would ensure healthy foods cost no more than the less healthy version, which refers to some foods where the nutritional quality can vary considerably between products, for example salt-reduced canned vegetables compared with standard canned vegetables, or fat-reduced cheese compared with full-fat cheese. Australia based Woolworths referred to developing an affordable healthy eating index. Whilst both initiatives show promise, transparency in determining the foods to monitor, criteria used to define ‘healthy’, impact on shopper behaviour, and actions to address unintended consequences are needed. Making data from these initiatives publically available to enable independent scrutiny would be of benefit to public health.

#### Food preferences and choices

Supermarkets committed to a variety of food labelling initiatives to assist consumers to identify foods that are: healthy or sustainable, meet specific needs, are locally sourced, or that address animal welfare concerns. Some supermarkets highlighted healthier foods using shelving signage or on their websites. The Guiding Stars scheme, implemented by Ahold Delhaize in the US and Loblaws in Canada, aims to overcome the plethora of packaging information by highlighting healthy choices using a shelf-edge tag and includes branded and own brand foods [85]. Guiding Stars has been effective in encouraging consumers to purchase more healthy foods [86]. A drawback of the Guiding Stars scheme is the lack of transparency in the algorithm applied to determine healthy foods, as it is a proprietary scheme [87]. This is important because nutrition ratings systems and symbols currently used around the world vary in their purpose and methods, achieving inconsistent dietary outcomes [88]. The benefit of supermarket-led whole-of-store schemes is that they remove the reliance on multiple manufacturers for implementation of voluntary front-of-pack labelling, facilitating widespread adoption and consumer use. Going forward, integrated assessment of environmental and nutritional factors is needed to promote healthy and sustainable food selection [89].

#### Nutritional quality

Nutrient targets for reformulation of processed own brand foods were referred to by some supermarkets. Whilst nutrient reduction policies of food manufacturers and retailers have been encouraged by many working in public health [28], others challenge this strategy, referring to it as ‘damage limitation’ [21], expressing concern that it may encourage consumption of ultra-processed foods [90]. Provision of own brand food ranges designated as healthy may assist consumers, however transparency of criteria used by supermarkets is needed to enable assessment.

Four supermarkets have shown leadership by setting targets for the nutritional quality of own brand food sold and two have extended this commitment to all food. These initiatives have great potential to hold supermarkets to account for their impact on population diets. Again, transparency of criteria to determine what constitutes healthy products is needed.

### Strengths and limitations

There are strengths and limitations to this study. A major strength is the systematic method adopted to select the world’s largest supermarkets, which means the CSR initiatives described have enormous scale and reach in the global population. This is the first study to summarise CSR commitments by global supermarkets that impact public health, which is important because of their governance role within the food system (whereby they influence policy and set rules). The number of countries affected by the selected supermarkets’ CSR actions demonstrates the global nature of their impact on public health. Limitations include the possibility that some important information was overlooked, as the research materials were restricted to reports that referred to CSR or sustainability for practical reasons. Supermarkets’ corporate websites may include additional information on their CSR actions, or provide some of the detail that was lacking in CSR reports, such as criteria applied to determine healthy products. Supermarkets were not contacted to provide further information or clarification as the purpose of the review was to examine publically available information. Quality of the statements made in supermarket reports was not evaluated as that was not the purpose of this descriptive analysis. It is recommended that further research is undertaken to explore these potential gaps and that quality should be considered in any future analysis of specific CSR commitments. The scope of this study did not include the ‘corporate political activity’ of global supermarkets (i.e. activity undertaken with the aim of influencing political outcomes that can impact public health, including lobbying and legal action [23]) which is an important gap in knowledge.

## Conclusions

The political CSR lens applied in this study identified the inherent responsibilities of powerful supermarkets to society, including food governance, the food system, and all aspects of a safe, nutritious and environmentally sustainable food system. CSR commitments made by 31 of the world’s largest supermarkets showed how they claim to support and encourage healthy and sustainable diets. Supermarkets’ efforts to demonstrate good corporate citizenship focused on: donating surplus food to charities to feed the hungry, reducing and recovering food waste, sustainably sourcing ingredients, governance of food safety, and growing their own brand foods. Although a number of supermarket CSR initiatives identified showed some progress is being made to address food waste, assure food safety and quality, and support selection of healthy foods, the world’s largest supermarkets could do more to use their power to support public health, including:

- Transparently report food waste encompassing the whole of the food system in waste reduction efforts;
- Support healthy and sustainable diets by reducing production and consumption of discretionary foods, meat, and other ingredients with high social and environmental impacts;
- Remove confectionery, sweetened beverages and nutrient poor snacks from prominent areas in stores;
- Ensure a variety of nutritious fresh and minimally processed foods are available; and
- Introduce initiatives that aim to make healthy foods more affordable, support consumers to select healthy and sustainable foods, and measure and report the proportion of healthy food sales as a proportion of total food sales, using transparent criteria for key terms.

## Abbreviations

ATNI: Access to nutrition index
CSR: Corporate social responsibility
GRI database: Global reporting initiative sustainability disclosure database
GRI: Global reporting initiative
INFORMAS: International network for food and obesity/noncommunicable diseases research, monitoring and action support
UN: United Nations
